# Losing the dogmatic view of cerebral autoregulation

**DOI:** 10.14814/phy2.14982

**Published:** 2021-07-28

**Authors:** Patrice Brassard, Lawrence Labrecque, Jonathan D. Smirl, Michael M. Tymko, Hannah G. Caldwell, Ryan L. Hoiland, Samuel J. E. Lucas, André Y. Denault, Etienne J. Couture, Philip N. Ainslie

**Affiliations:** ^1^ Department of Kinesiology Faculty of Medicine Université Laval Québec QC Canada; ^2^ Research center of the Institut universitaire de cardiologie et de pneumologie de Québec Québec QC Canada; ^3^ Sport Injury Prevention Research Centre Faculty of Kinesiology University of Calgary Calgary AB Canada; ^4^ Cerebrovascular Concussion Laboratory Faculty of Kinesiology University of Calgary Calgary AB Canada; ^5^ Human Performance Laboratory Faculty of Kinesiology University of Calgary Calgary AB Canada; ^6^ Hotchkiss Brain Institute University of Calgary Calgary AB Canada; ^7^ Integrated Concussion Research Program University of Calgary Calgary AB Canada; ^8^ Alberta Children’s Hospital Research Institute University of Calgary Calgary AB Canada; ^9^ Libin Cardiovascular Institute of Alberta University of Calgary AB Canada; ^10^ Neurovascular Health Laboratory University of Alberta Edmonton AB Canada; ^11^ Center for Heart, Lung and Vascular Health School of Health and Exercise Sciences University of British Columbia – Okanagan Kelowna BC Canada; ^12^ Department of Cellular and Physiological Sciences Faculty of Medicine University of British Columbia Vancouver BC Canada; ^13^ Department of Anesthesiology, Pharmacology and Therapeutics University of British Columbia Vancouver BC Canada; ^14^ School of Sport, Exercise and Rehabilitation Sciences College of Life and Environmental Sciences University of Birmingham Birmingham United Kingdom; ^15^ Centre for Human Brain Health University of Birmingham Birmingham United Kingdom; ^16^ Department of Anesthesiology and Critical Care Division Montreal Heart Institute Montreal QC Canada; ^17^ Division of Critical Care Medicine Centre Hospitalier de l’Université de Montréal Montreal QC Canada

**Keywords:** arterial blood pressure, autoregulatory curve, cerebral autoregulation, cerebral blood flow, Lassen

## Abstract

In 1959, Niels Lassen illustrated the cerebral autoregulation curve in the classic review article entitled *Cerebral Blood Flow and Oxygen Consumption in Man*. This concept suggested a relatively broad mean arterial pressure range (~60–150 mmHg) wherein cerebral blood flow remains constant. However, the assumption that this wide cerebral autoregulation plateau could be applied on a within‐individual basis is incorrect and greatly variable between individuals. Indeed, each data point on the autoregulatory curve originated from independent samples of participants and patients and represented interindividual relationships between cerebral blood flow and mean arterial pressure. Nonetheless, this influential concept remains commonly cited and illustrated in various high‐impact publications and medical textbooks, and is frequently taught in medical and science education without appropriate nuances and caveats. Herein, we provide the rationale and additional experimental data supporting the notion we need to lose this dogmatic view of cerebral autoregulation.

## Graphical abstract

In 1959, Niels Lassen illustrated the cerebral autoregulation curve in the classic review article entitled Cerebral Blood Flow and Oxygen Consumption in Man. This concept suggested a relatively broad mean arterial pressure range wherein cerebral blood flow remains constant. Herein, we provide the rationale and additional experimental data supporting the notion we need to lose this dogmatic view of cerebral autoregulation.

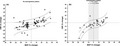

## 1 EARLY DESCRIPTION OF THE CEREBROVASCULAR PRESSURE–FLOW RELATIONSHIP

The early understanding and description of the cerebrovascular pressure–flow relationship, as described by Roy and Sherrington in 1890, who examined changes in brain volume or cerebral venous pressure following arterial pressure changes in diverse animal models (i.e., dogs, cats, and rabbits) (Roy & Sherrington, 1890), was defined as: “the higher the arterial pressure, the greater is the amount of blood which passes through the cerebral blood‐vessels and vice versa; and, so far as we have been able to learn, this law holds good for all changes in the arterial pressure whatever be their cause” (Roy & Sherrington, 1890). Shortly thereafter in 1895, Bayliss et al. (1895) also noted in dogs that metabolic‐induced elevations in arterial pressure were reflected in near simultaneous and reciprocal changes in cerebral venous pressure [as an index of cerebral blood flow (CBF)]. These authors concluded that “In all physiological conditions a rise of arterial pressure accelerates the flow of blood through the brain, and a fall slackens it” (Bayliss et al., 1895). Although CBF was not directly measured in these classical experiments, these definitions highlighted the potential “pressure‐passive” nature of the cerebral vasculature. Then, in the early 1930s, Fog provided evidence of rapid diameter changes of pial arteries and arterioles induced by changes in arterial pressure in anesthetized cats (Fog, 1938), suggesting the presence of an arterial pressure range wherein autoregulation seemed effective.

## 2 LASSEN'S CEREBRAL AUTOREGULATION CURVE

**FIGURE 1:**
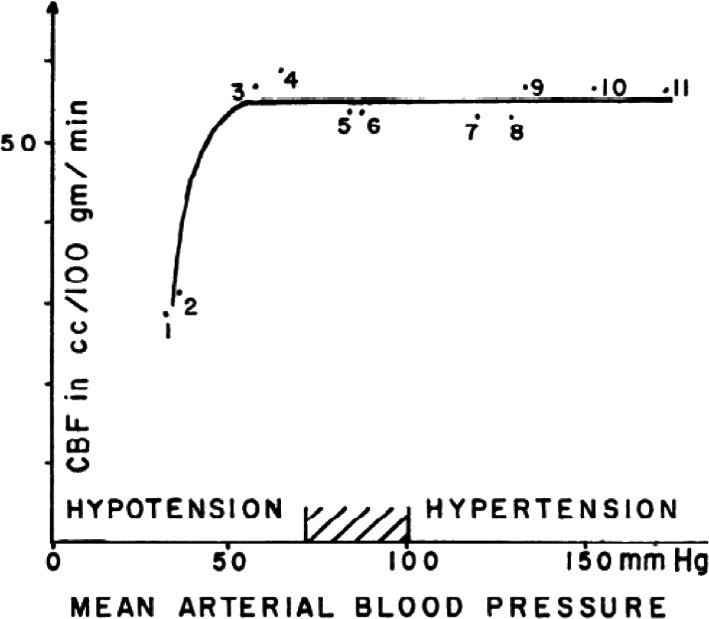
Cerebral blood flow and blood pressure. Mean values of 11 groups of subjects reported in seven studies have been plotted. Various acute and chronic conditions have been selected, characterized by a change in blood pressure. See text for details. Reprinted from Lassen (1959) with permission

**FIGURE 2:**
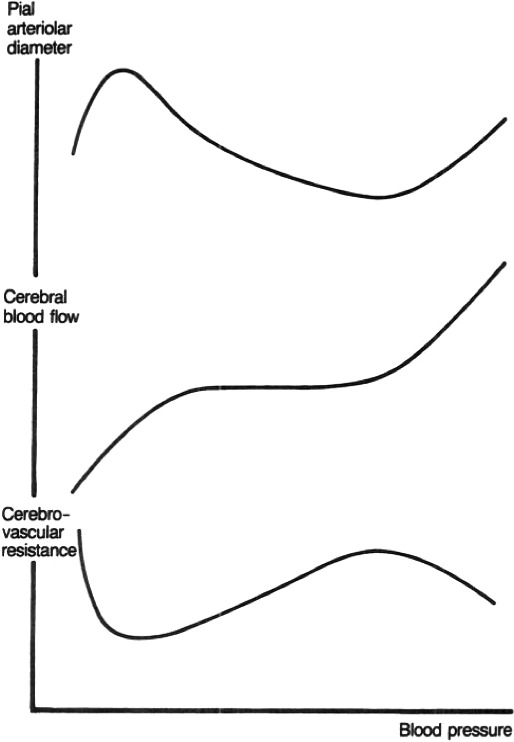
A diagrammatic representation of the relationship of pial arteriolar diameter, cerebral blood flow, and cerebrovascular resistance with blood pressure. It will be seen that pial arteriolar dilatation reaches a maximum well below the lower level of autoregulation. Reprinted from Paulson et al. (1990) with permission

These early views were accepted for more than 60 years as to how CBF was regulated until the cerebral autoregulation curve was presented by Niels Lassen (Figure 1) in the classic review paper entitled “Cerebral Blood Flow and Oxygen Consumption in Man” (Lassen, 1959). This work suggested a relatively wide mean arterial pressure range (~60–150 mmHg) wherein CBF remained constant (~50 ml/min/100 g brain mass). The assumption Lassen's curve could be applied on a within‐individual basis has since been determined as erroneous and highly variable between individuals (Drummond, 1997). Indeed, each data point on the curve originated from independent populations of volunteers and patients and represented interindividual relationships between CBF and mean arterial pressure. Lassen constructed the curve based on averaged or adjusted CBF and mean arterial pressure measurements in 376 individuals across seven different studies involving 11 widely varied groups. For instance, these various states of mean arterial pressure were established in otherwise healthy humans via administration of highly vasoactive drugs (i.e., hexamethonium, veratrum viride, apresolin, norepinephrine, aramine) and also in pathological systemic hypertension (i.e., hypertensive toxemic pregnancy, essential hypertension). Overall, these diverse and clinically distinct data‐sets were employed to construct the well‐known mean arterial pressure plateau region that has been associated with a “constant” CBF across a very wide range of cerebral perfusion pressure (mean arterial pressure – intracranial pressure) (Lassen, 1959) (Figure 1). In 1990, Paulson updated the “classical curve” to include an upper limit of the autoregulatory curve based on data published in an animal model (cats; Figure 2) (Paulson et al., 1990).

This influential concept has been regularly cited since its appearance and has been illustrated in numerous high‐impact publications and textbooks (Barrett et al., 2010; Dagal & Lam, 2009; Patel et al., 2019). Also, this traditional view is routinely taught, often without nuances or caveats, in medical and science education. It should be acknowledged that Lassen was, without question, a modern‐day pioneer in the field of cerebrovascular physiology with endless unique contributions to advancing the measurement of CBF as well as the related physiological and pathophysiological implications [reviewed in: (Paulson & Parving, 2007; Severinghaus, 1999)]. Although not a universal finding, we also appreciate some experimental data from studies using preclinical models (e.g., cats (Faraci et al., 1987; Kontos et al., 1978; MacKenzie et al., ,^1976^, ^1979^), rats (Harper & Bohlen, 1984; Harper et al., 1984; Hernandez et al., 1978; Hoffman et al., 1991; Sadoshima & Heistad, 1983), baboons (Fitch et al., 1976a, 1976b; Harper et al., 1973; Strandgaard et al., ,^1974^, ^1976^), monkeys (Heistad et al., 1980), rabbits (Tureen et al., 1990)) broadly support Lassen's interpretation of cerebral autoregulation—providing that the change in arterial pressure is slow. What we report and discuss herein represents a natural progress in science as a result of several years of advanced insights and multiples modes of imaging CBF. In fact, if Lassen have had access to such advanced monitoring tools, his interpretation of cerebral autoregulation might have been very different. Over the past 60+ years, our physiological understanding of the relationship between CBF and arterial pressure has evolved well beyond the concepts originally presented by Lassen (1959).

This “classical view” of cerebral autoregulation has since been disputed, challenged for years, and essentially disproven (Drummond, 2019; Heistad & Kontos, 1983; Willie et al., 2014). We are thus not the first—and certainly not the last—group of researchers and clinicians to highlight and discuss these misconceptions and over interpretation of Lassen's traditional cerebral autoregulation curve. However, considering (1) the amount of actual literature in the cerebrovascular physiology field still introducing the cerebral autoregulation concept solely using Lassen's curve without appropriate nuances, (2) the potential clinical implications of using such a framework to guide the clinical management of cerebral perfusion pressure, as well as (3) the evidence available on cerebral autoregulation, there is a critical need to revisit once again these important theoretical concepts. This update is especially important for those who base some of their clinical management decisions on this traditional autoregulatory curve. In this perspective paper, not all aspects of cerebral autoregulation will be extensively covered. The reader is invited to read the excellent comprehensive review article by Claassen et al. (2021) for a complete understanding of physiological and clinical implications of cerebral autoregulation. Rather than repeat this information, we outline an explicit rationale, and provide new additional experimental data which, together, support the view to move away from the traditional view of cerebral autoregulation.

## 3 CEREBRAL AUTOREGULATION: BACK TO BASICS

The brain is one of the most highly perfused organs in the human body; its weight is less than 2% of body mass but it receives 15 to 20% of resting cardiac output (Williams & Leggett, 1989), and hence, high proportion of resting oxygen consumption. There is also a lack of oxygen reserve within the brain. Yet, the inflexible structure surrounding the brain—the skull—allows for only very minor changes in cerebrospinal fluid and/or tissue expansion. For these reasons, it is therefore of utmost importance to have tightly regulated mechanism(s) to maintain a relatively constant CBF, and thus, cerebral blood volume. By the end of the 1800s, the foundation of our early comprehension of some of these mechanism(s) regulating CBF had been established, and included primarily: (1) the Monro–Kellie doctrine (Abercrombie, 1836) (which has been challenged and updated since its inception (Kalisvaart et al., 2020; Wilson, 2016)); (2) the notion cerebral metabolism influences CBF (Mosso, 1880; Roy & Sherrington, 1890); and (3) perturbations to the systemic circulation are detected in the cerebral circulation (Bayliss et al., 1895).

**FIGURE 3:**
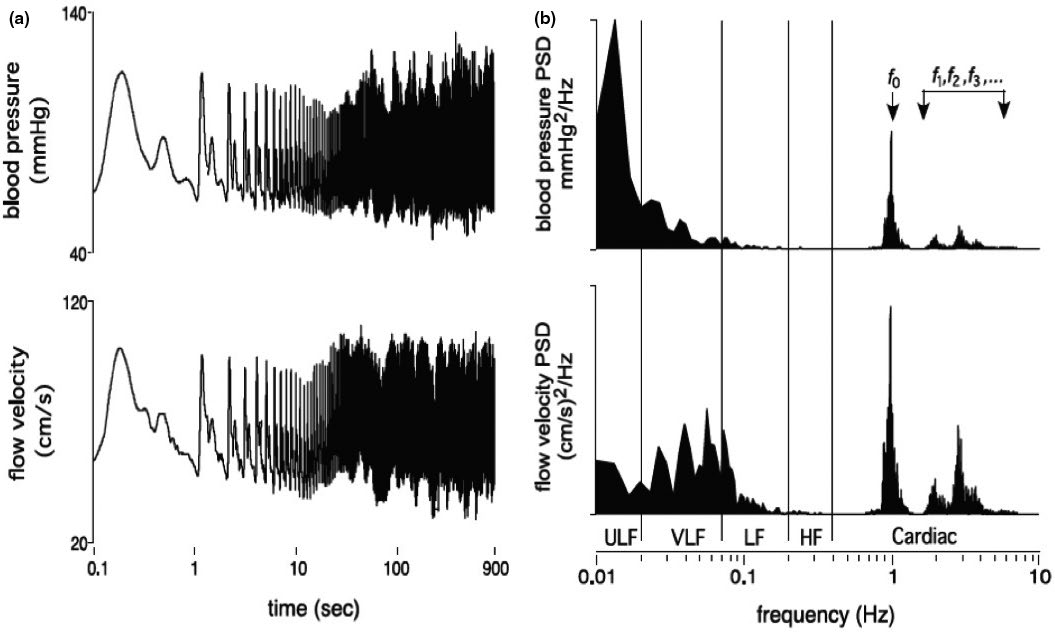
(a) Finger arterial blood pressure and middle cerebral blood flow velocity over 900 s (presented on log axes) for a human subject in the seated resting position. (b) The corresponding power spectrums, which decompose the time series signals into its various constituent component frequencies. Note that the fundamental frequency (*f*_0_) and its harmonics (*f*_1_, *f*_2_,*f*_3_) correspond to pulsations coincident with the pulse, whereas progressively lower frequency components reflect the longer term oscillations and trends in the time domain. *ULF* ultra‐low frequency, *VLF* very low frequency, *LF* low frequency, *HF* high frequency. Reprinted from Tzeng & Ainslie (2013) with permission

One of the key regulators of CBF is therefore mean arterial pressure via cerebral autoregulation (Willie et al., 2014); this can be defined as the intrinsic ability of the brain to independently regulate CBF, via changes in cerebrovascular resistance, when arterial blood pressure fluctuates (see: Figure 2). Cerebral autoregulatory mechanisms remain, however, poorly understood, especially in human. This is likely a result of the “black‐box” nature of the multifactorial and redundant pathways responsible for CBF regulation (Willie et al., 2014), as well as the different methods to assess CBF or difference in physiological stress elicited by diverse experimental models used to examine these autoregulatory mechanisms (Tzeng & Ainslie, 2013). While cerebral autoregulation is typically categorized as having both static and dynamic components, this categorization is an experimental construct rather than a physiological distinction per se. For example, static cerebral autoregulation is classically described as functioning throughout several minutes to hours and denotes the *steady*‐*state* relationship between mean arterial pressure and CBF. In contrast, dynamic cerebral autoregulation generally refers to the cerebral pressure–flow relationship as observed *during transient changes* (over a period of seconds) in mean arterial pressure (Tzeng & Ainslie, 2013) (Figure 3). Rather than separate physiological identities, however, it is becoming clearer these two concepts are merely a continuum of one another. Static cerebral autoregulation reflects the ultra‐low frequency oscillations (0–0.02 Hz or >50 seconds to complete one oscillation) and dynamic cerebral autoregulation reflects the very low frequency (0.02–0.07 Hz or 50.0 to 14.3 seconds to complete one oscillation), low‐frequency (0.07–0.20 Hz or 14.3 to 5.0 seconds to complete one oscillation), and high‐frequency (0.20–0.35 Hz 5.0 to 2.9 seconds to complete one oscillation) range oscillations (Claassen et al., 2016; Tzeng & Ainslie, 2013; Zhang et al., 1998). Under normal physiological conditions, frequencies above 0.20 Hz have been shown to occur at a greater rate than are able to be dampened by cerebral autoregulatory properties and are often considered to be outside of the cerebral autoregulation realm (Claassen et al., 2016). One key issue with Lassen's cerebral autoregulatory curve thus lies in its overinterpretation, as well as the extrapolation of the findings presented in his review article. Indeed, one cannot extrapolate data gathered from scenarios where slow and between‐individual steady‐state changes in arterial pressure were used to build the cerebral autoregulatory curve, to a physiological/clinical scenario where rapid within‐individual surges/fluctuations in arterial pressure are examined. Several studies focusing on dynamic cerebral autoregulation clearly show CBF can fluctuate tremendously, even if static cerebral autoregulation is unimpaired (in healthy participants) (Aaslid et al., ,^1989^, ^2007^; Labrecque et al., 2021, 2017, 2019; Lind‐Holst et al., 2011; Tzeng et al., 2010). In other words, sudden changes in mean arterial pressure are transmitted directly to the cerebral circulation, although CBF tends to return to its baseline value within a brief time period.

## 4 LASSEN'S CEREBRAL AUTOREGULATORY CURVE: DISPUTED VIEW OF CEREBRAL AUTOREGULATION

The autoregulatory curve presented by Lassen has been challenged by several experts in the field. In 1983, Heistad & Kontos (1983) elegantly disputed the concept that CBF remains constant notwithstanding changes in mean arterial pressure. They presented compelling evidence revealing some of the points were acquired with drugs known to directly increase CBF during systemic hypotension (hydralazine or veratrum alkaloids). Such pharmacological interventions are known to cause dilation of cerebral vessels and likely alter cerebral autoregulation properties via direct alterations in cerebrovascular tone. Other points included in the plateau region did not correspond to data from the original cited studies (i.e., Lassen mistakenly reduced the original data by ~20% in order to have the data better fit the “curve”). In the three studies comprised within Lassen's curve where mean arterial pressure was altered, CBF was noted to also change even with minor alterations in arterial pressure (~7% change in CBF per 10 mmHg in arterial pressure). Another important issue is cerebral perfusion pressure should be monitored when examining cerebral autoregulation. Considering intracranial pressure needs invasive monitoring, although recent work in patients with traumatic brain injury suggests intracranial pressure can also be non‐invasively measured on a beat‐by‐beat basis (Frigieri et al., 2018), cerebral perfusion pressure will usually be substituted by mean arterial pressure, assuming no intracranial hypertension. However, cerebral perfusion pressure can also be influenced by changes in central venous pressure or high intracranial pressure in specific physiological conditions (e.g., postural change) or pathologies (e.g., right ventricular dysfunction).

## 5 LASSEN'S CEREBRAL AUTOREGULATION CURVE: WELL ANCHORED IN THE CLASSROOM AS WELL AS IN THE OPERATING ROOM

Since the publication of Lassen's autoregulatory curve (Lassen, 1959) [and the update by Paulson et al. (1990)], clinicians have often been applying this traditional framework to manage mean arterial pressure (and thus cerebral perfusion pressure) of their patients in different clinical situations (e.g., to correct anesthesia‐induced systemic hypotension or to maintain mean arterial pressure above what is considered the lower limit of cerebral autoregulation during the cardiopulmonary bypass of a cardiac surgery) aimed at reducing cerebrovascular complications. Nowadays, the concept proposed by Lassen remains included in prominent textbooks. For instance, although updated in the most recent edition of Miller's Anesthesia (9th edition, 2019), a standard reference work in anesthesiology, the following “key point” still appears in the “Cerebral Physiology and the Effects of Anesthetic Drugs” chapter: “CBF is autoregulated and remains constant over a mean arterial pressure (MAP) range estimated at 65 to 150 mmHg, given normal venous pressure. CBF becomes pressure passive when MAP is either less than the lower limit or more than the upper limit of autoregulation. The lower and upper limits, as well as the range and slope of the plateau, manifest significant variability between individuals” (Patel et al., 2019). However, this management of mean arterial pressure is performed assuming the averaged data and theoretical illustration proposed by Lassen (1959) can be applied on a within‐individual basis, a practice that has been questioned for years (Drummond, 1997). Yet, many health care professionals still refer to Lassen's curve when utilizing the collection of physiologic data necessary to quantify cerebral autoregulation and individualized arterial pressure targets (Joshi et al., 2010, 2012; Ono et al., 2012; Zeiler et al., 2019). This strategy allows the identification of an “optimal” cerebral perfusion pressure or mean arterial pressure, that is an individualized threshold at which cerebral autoregulation is thought to be the most effective. In theory, this optimal value indicates a point between the lower and upper limit of cerebral autoregulation on the Lassen's curve (Zeiler et al., 2019). However, time spent away from this optimal value is associated with adverse clinical outcomes (Jaeger et al., 2010; Joshi et al., 2010, 2012; Ono et al., 2012; Sekhon et al., 2019; Zeiler et al., 2019), arguing against a wide range of cerebral perfusion pressure/mean arterial pressure where CBF remains constant.

## 6 MOVING FROM A TRADITIONAL VIEW TOWARD A CONTEMPORARY UNDERSTANDING OF CEREBRAL AUTOREGULATION

**FIGURE 4:**
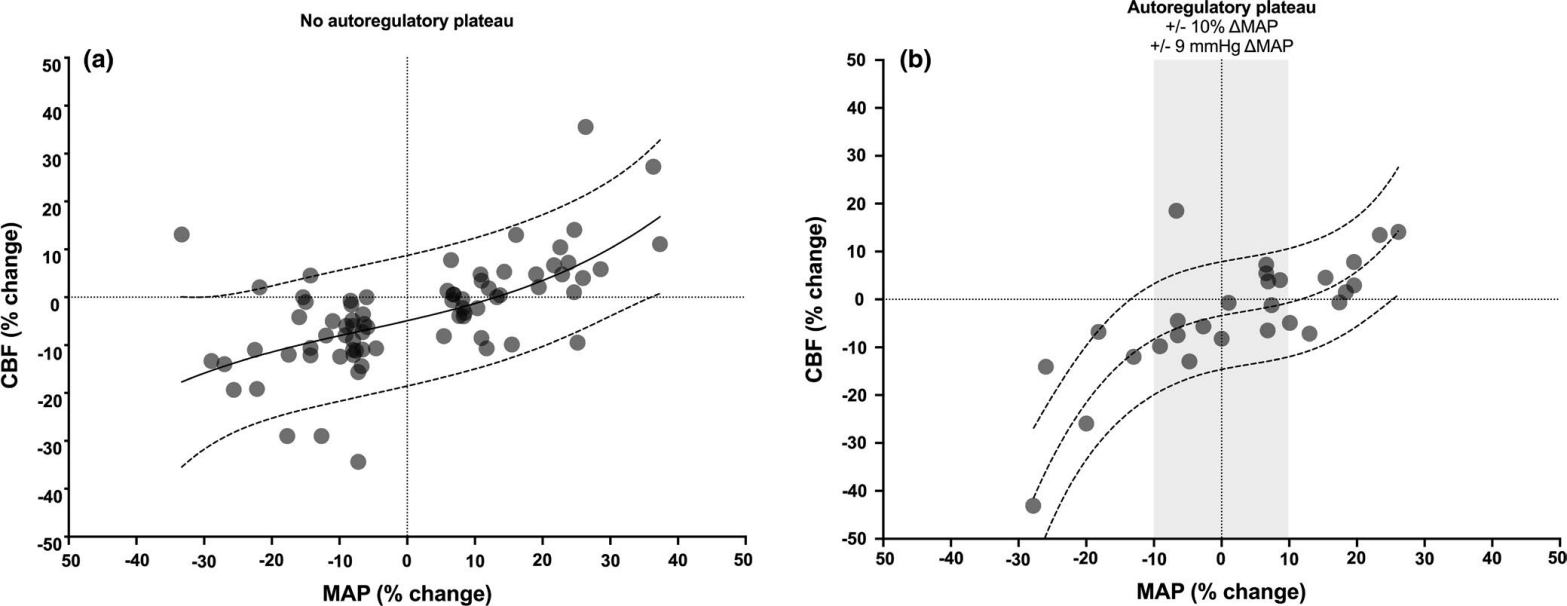
Relationship between relative changes in MAP from baseline (Δ%MAP) and concomitant relative changes in CBF (Δ%CBF) when MAP decreases and increases including (left panel) and excluding (right panel) the utilization of cardiovascular drugs to manipulate MAP. These data represent an updated analysis initially published by Numan et al. (2014) Note the consistent pressure‐passive nature of CBF in the pharmacological data (left panel) and the small plateau present in the non‐pharmacological data (right panel)

Since the previous challenge by Heistad & Kontos (1983), accumulating findings continue to confront the Lassen's view of the relationship between mean arterial pressure and CBF and the presence of a wide cerebral autoregulatory plateau remains questioned (Rosenblum, 1995). For example, at least in otherwise healthy and non‐anesthetized humans, recent evidence now indicates the range of the mean arterial pressure plateau is considerably smaller (~5–10 mmHg) than originally proposed where CBF is stable (Tan, 2012; Tan et al., 2013), as compared to the ~90 mmHg range proposed by Lassen (1959). In support of this argument, we present herein additional data from an updated analysis initially published by Numan et al. (2014). In the original analysis by these authors (Numan et al., 2014), a Pubmed search resulted in a total of 459 studies. The exclusion procedure (see flowchart presented in figure 1 of Numan et al. (2014)), led to inclusion of 49 experiments (23 experiments where MAP was decreased and 26 experiments where MAP was increased). For the current updated analysis, in addition to the dataset from Numan et al. (2014), Pubmed was searched for studies with the terms “arterial pressure,” “cerebral blood flow,” and “healthy subjects,” having been published between January 2012 and November 2020. Non‐human experiments and non‐English studies were excluded. As presented in Numan et al. (2014), the selected population was healthy participants between 18 and 65 years. Within studies which included both patients and control participants, the results of the control participants were included and the results of the patients were excluded. All other inclusion and exclusion criteria reported by Numan et al. (2014) were also replicated. The updated examination resulted in the review of an additional 181 studies. The exclusion procedure led the inclusion and addition of seven experiments (four where MAP was decreased and three where MAP was increased) to the original dataset of Numan et al. (2014) Accordingly, the current analysis included 27 experiments where MAP decreased and 29 experiments where MAP increased. A 3^rd^ order polynomial function (to maximize goodness of fit) was utilized to analyze this dataset and outliers (three data points in left panel and two data points in right panel of Figure 4) were identified and removed from fits using the ROUT method (with Q set to 1%). Findings presented in Figure 4 thus represent relative changes in MAP and CBF from baseline, and show the cerebral autoregulation plateau is considerably smaller than originally proposed. This observation can be appreciated especially with data from the right panel of Figure 4, where any pharmacological interventions (some of which likely alter cerebral autoregulation properties via direct alterations in cerebrovascular tone) were excluded from these analyses.

It is thus not surprising that the majority of the research focusing on the lower and upper limits of cerebral autoregulation—assuming they even exist—indicates these limits widely vary between individuals and across disease‐states (Drummond, 1997). Even modest steady‐state or dynamic changes in mean arterial pressure (~10–15 mmHg; Figure 4) influence CBF. Thus, CBF (or a surrogate of CBF such as cerebral blood velocity or tissue oxygenation), in addition to mean arterial pressure, need to be closely monitored in clinical situations where cerebral perfusion is at risk (i.e., general anesthesia, deliberate or unplanned hypotension [tilt testing, postural changes], nerve blocks during shoulder surgery in a beach chair position, etc.). Another important physiological issue related to cerebral autoregulation, which was not taken into consideration in the classic autoregulation curve, is the recently appreciated presence of ‘asymmetry’ in the cerebral autoregulation response (Numan et al., 2014). Indeed, our group and others have presented compelling evidence the human brain seems better adapted to compensate for transient increases (compared to decreases) in mean arterial pressure, based on findings comparing steady‐state (Numan et al., 2014) or dynamic changes in mean arterial pressure in healthy volunteers (Brassard et al., 2017; Labrecque et al., 2021; Panerai et al., 2018; Tzeng et al., 2010) or patients with head injuries (Aaslid et al., 2007). In addition, the cerebrovasculature is also more efficient at buffering changes in blood pressure associated with the systolic aspect of the cardiac cycle (Burma et al., 2020; Smirl et al., 2018). While further research is needed to describe this phenomenon in other populations and additional clinical conditions, this observation is highly relevant for various physiological (e.g., rapid eye movement sleep, exercise), and pathological clinical situations (e.g., uncontrolled systemic hypertension, autonomic dysreflexia) necessitating an efficient counter‐regulation in CBF against transient mean arterial pressure surges. Importantly for the clinician, this directional sensitivity of brain vessels to changes in mean arterial pressure should also be integrated in the framework to guide the management of mean arterial pressure in the operating room. For instance, during surgeries which include deliberate systemic hypotension where mean arterial pressure is maintained at what is parochially considered to be the lower limit of cerebral autoregulation. Therefore, in these common clinical situations, the brain is likely at risk of hypoperfusion, because of this reduced ability of cerebral vessels to efficiently buffer large reductions in mean arterial pressure; as such, these situations are associated with some of the poor neurologic outcomes following these situations (Selnes et al., 2012).

## 7 METHODOLOGICAL CONSIDERATIONS

It should be noted that several attempts to characterize the within‐individual relationship between CBF and mean arterial pressure have been hindered by normal physiological and reflex responses of the healthy human body. For example, in vivo human models show the arterial baroreflex functions to limit the mean arterial pressure range that can be investigated. This represents a major issue for characterizing and interpreting static cerebral autoregulation. Indeed, it impedes our ability to maintain and isolate static changes in arterial pressure in humans with intact baroreflex responses. Alternatively, vasoactive drugs (i.e., nitroglycerin, nitroprusside, calcium channel blocker, hydralazine, phenylephrine, norepinephrine, ephedrine and indomethacin) or physical manipulations leading to central blood volume shifts (i.e., head‐up tilt, severe lower body negative pressure) can be used to characterize static cerebral autoregulation. However, some of these methods to manipulate mean arterial pressure have the ability to influence CBF, independently from cerebral autoregulation, via direct effects on tone or indirect effects on arterial carbon dioxide tension or cerebral metabolism. In addition, the effects that some anesthetic agents have on CBF (i.e., isoflurane and sevoflurane can increase CBF while propofol can reduce CBF (Slupe & Kirsch, 2018)) will also confound interpretation of cerebrovascular reflexes per se in the operating room. Transcranial Doppler ultrasound has been regularly used in studies examining CBF regulation and cerebral autoregulation (particularly dynamic cerebral autoregulation) for many reasons (Claassen et al., 2016). This technology is appealing considering its portability and non‐invasiveness and its ability to obtain uninterrupted and high‐frequency data. Transcranial Doppler ultrasound monitors blood velocity in intracranial arteries (e.g., middle cerebral artery) and not volumetric flow. Transcranial Doppler ultrasound measurements can indeed only be representative of (regional) CBF if the diameter of the artery of interest remains constant (Willie et al., 2011). Recent experimental work shows conditions such as hypocapnia, hypercapnia, hypoxia, and handgrip exercise can induce changes in vessel diameter, leading to blood flow measurements errors (Coverdale et al., 2014; Hoiland et al., 2015; Mikhail Kellawan et al., 2017; Verbree et al., 2014, 2016). However, if studies are carefully planned, this limitation can be minimized (Ainslie & Hoiland, 2014). In addition, this limitation could certainly be overcome by simultaneously examining global CBF, by combining volumetric flow (or diameter) in internal carotid artery and vertebral artery utilizing Duplex ultrasound (Hoiland et al., 2020; Smith et al., 2014; Thomas et al., 2015). Of importance, the typical message coming from magnetic resonance imaging studies is transcranial Doppler ultrasound measurements are not providing precise/accurate CBF estimation (using magnetic resonance imaging as the reference); therefore, transcranial Doppler ultrasound may not represent accurate CBF due to the possibility of the insonated intracranial artery changing in diameter in response to carbon dioxide changes, handgrip exercise, etc (Ainslie & Hoiland, 2014; Coverdale et al., 2014; Hoiland & Ainslie, 2016; Verbree et al., 2014, 2016). Usually, magnetic resonance imaging studies use predefined regions of interests, or other forms of magnetic resonance imaging (e.g., T1, T2, VIPR, time of flight methods, etc.) approaches, which allow quantification of vascular dimension or blood flow through specific arteries. This being acknowledged, in addition to the various assumptions of the above‐mentioned approaches, these are still rather different from identifying areas of elevated CBF per se. Also, while one can argue magnetic resonance imaging could overcome transcranial Doppler ultrasound‐related limitations by examining global CBF, this approach has poor temporal resolution to monitor rapid CBF associated with dynamic cerebral autoregulation quantification. Another significant limitation of magnetic resonance imaging is participants can only be examined while in supine position, which limits experimental conditions to quantify cerebral autoregulation. We thus consider neither magnetic resonance imaging nor transcranial Doppler ultrasound can be assumed as a standard for measuring CBF. Despite these methodological limitations, the available literature describing the within‐individual relationship between CBF and mean arterial pressure provide a substantial basis that indicates blood flow in the brain is not constant over an extended range of mean arterial pressure (Tan, 2012; Tan et al., 2013; Tzeng & Ainslie, 2013; Willie et al., 2014).

## 8 CONCLUSION

Considering the compelling evidence currently available, it is now time for the medical community to move away from the commonly touted view of cerebral autoregulation presented in “Cerebral Blood Flow and Oxygen Consumption in Man” (Figure 1). As such, we urge educators to update how they teach this crucial concept to medical professionals. This educational concept should not be achieved only by focusing without nuances on the traditional cerebral autoregulation curve suggested by Lassen, but also by employing the contemporary data indicating CBF regulation is far more pressure‐passive in nature than traditionally believed (Figure 4). While a small cerebral autoregulatory plateau region may exist wherein CBF will remain relatively constant in response to slower steady‐state changes in mean arterial pressure (e.g., infusion of a vasoactive drug), CBF will not necessarily remain stable in all physiological/clinical conditions. Indeed, CBF will fluctuate or may even become (almost) pressure passive in other conditions related to faster arterial pressure changes (e.g., repeated squats, acute exercise, sit‐to‐stand). One key point is that in the same individual/patient who can tolerate a large but slow change in arterial pressure without a significant change in CBF, a similar but much faster change in arterial pressure can importantly reduce (or increase) CBF. Additionally, it should be observed that the blood vessels of the brain appear to be more effective at protecting the microcirculation against steady‐state and transient increases, compared to decreases, in mean arterial pressure (Aaslid et al., 2007; Brassard et al., 2017; Numan et al., 2014; Panerai et al., 2018; Tzeng et al., 2010). For example, in clinical situations such as surgical procedures including deliberate blood pressure reductions maintained at what is considered to be the lower limit of cerebral autoregulation, the brain is likely at risk of hypoperfusion, because of this attenuated ability of the cerebrovasculature to efficiently dampen large reductions in mean arterial pressure. Accordingly, this directional sensitivity of the cerebral vessels to changes in mean arterial pressure should thus also be referred to when introducing the concept of cerebral autoregulation in the scientific literature and medical school's courses, and incorporated in the framework to guide the management of mean arterial pressure in the operating room.

## CONFLICT OF INTEREST

The authors declare no conflict of interest.

## AUTHORS CONTRIBUTIONS

P.B. contributed to the original idea of the review article; L.L. analyzed the data; P.B., L.L., and P.N.A. interpreted the results; L.L. prepared Figure 4; P.B. drafted the article; L.L., J.D.S., M.M.T., H.G.C., R.L.H., S.J.E.L, A.Y.D., E.J.C., and P.N.A. edited and revised the manuscript; all authors approved the final version of manuscript.
